# 

**Published:** 1843-07-08

**Authors:** 


### CONTENTS.

Brief remarks on the treatment of fistula lacrymalis by dilatation. By Isaac Parrish, M. D. - - - - - 145
Case of abscess through the lumbar region of the back—discharge of twenty stones. By A. H. Perkins, M. D. - 146
Case of poisoning by laudanum. By J. L. Burt, M. D. - - - - - 146
Acetic extracts, - - - - - 146
*Jefferson Medical College.*
Clinic of Professor Mitchell, - - 147
Philadelphia Hospital, service of Meredith Clymer, M. D. Case of soft cancer of the ovary, with a multilocular cystic dropsy, - - - - 148
*Bibliographical Notices.*
The kidneys and urine. By J. J. Berzelius. Translated from the German, by M. H. Boyé and F. Leaming, - 149
A practical treatise on the management and diseases of children. By Richard G. Evanson, M. D., Professor of Medicine in the Royal College of Surgeons, Ireland, and H. Maunsell, M. D., Professor of Political Medicine in the Royal College of Surgeons, Ireland. Second American, from the fourth Dublin edition, with notes, by D. Francis Condie, M. D., Fellow of the College of Physicians of Philadelphia, &c., - - - - - - 149
New work by Dr. F. Campbell Stewart, 149
Onychia maligna of the great toe. By A. Colles, M. D. - - - - 150
Influence of season on the production of pneumonia in children, - - 150
*Retrospect of the Medical Sciences.*
Treatment of pneumonia, - - - 151
Somnambulic dogs, - - - - 151
Cold infusion of cinchona cordifolia, - 151
A hint to magnetisers, - - - 152
Comparative weight of the human brain in the male and female, - - - 152
Prognosis of pneumonia, - - - 152
Extract of a lecture on syphilis. By Dr. Philip Ricord, - - - - 152
Indications afforded by the state of the iris in cases of cerebral lesion, - 154
On the comparative frequency of the morning and evening pulse. By Dr. Stratton, - - - - - - 154
Results of homœopathy, - - - 155
Analysis of the blood in six cases of morbus brightii, - - - 155
Compound fracture of leg, - - - 155
Treatment of tinea favosa, - - - 155
Observations on the microscopic characters and manner of developement of the blood corpuscles. By Dr. M. Barry, 156
Typhoid fever amongst animals, - 156
Turning of plants toward the light, - 156
Influence of asphyxia on the secretion of bile, - - - - - - - 156
Sulphate of quinine in rheumatism, - 156

